# Clinical importance of second-opinion interpretations by radiologists specializing in gynecologic oncology at a tertiary cancer center: magnetic resonance imaging for endometrial cancer staging

**DOI:** 10.1590/0100-3984.2016.0171

**Published:** 2018

**Authors:** Inês Alves, Teresa Margarida Cunha

**Affiliations:** 1 MD, Department of Radiology, Hospital Central do Funchal, Funchal, Portugal.; 2 MD, Department of Radiology, Instituto Português de Oncologia de Lisboa Francisco Gentil, Lisboa, Portugal.

**Keywords:** Endometrial neoplasm/diagnostic imaging, Referral and consultation, Magnetic resonance imaging, Diagnostic imaging, Tertiary care centers

## Abstract

**Objective:**

To determine whether there are substantive differences between the initial interpretations of magnetic resonance imaging (MRI) scans acquired at outside facilities and the second-opinion interpretations of radiologists specializing in gynecologic oncology at a tertiary cancer center, among patients referred for endometrial cancer staging.

**Materials and Methods:**

This was a retrospective, comparative analysis of 153 initial and second-opinion MRI reports for endometrial cancer staging officially submitted for review by radiologists specializing in gynecologic oncology. For each case, the relationship between the initial and second-opinion reports, regarding the suggested diagnosis and the clinically relevant MRI findings reported, was categorized as "agreement" or "disagreement". Histopathology was used in order to establish the definitive diagnosis.

**Results:**

Disagreement was found in 58 (37.9%) of the 153 cases. Second-opinion interpretations reported findings that affected the preoperative cancer staging and could have led to a change in treatment in 38 cases (24.8%); that did not affect the preoperative staging but provided information that was more accurate in 8 (5.2%); and that suggested a new cancer diagnosis in 12 (7.8%). In 37 cases (24.2%), there was a potential for changes in patient care. Among the 58 cases of disagreement, a definitive (histopathological) diagnosis was made in 41 (70.7%). In 31 (75.6%) of those 41 cases, the second-opinion report was more accurate than was the initial report.

**Conclusion:**

Discordant interpretations of MRI examinations, which can have a substantial effect on the clinical management of patients, appear to be common.

## INTRODUCTION

As at many other tertiary care centers, patients referred to our hospital for definitive evaluation and treatment have already undergone multiple imaging examinations at outside institutions^(^^1^^)^. In the particular case of our gynecology department, a second-opinion interpretation of those imaging examinations by a radiologist specializing in gynecologic oncology is frequently requested. When the second-opinion report is being formulated, the radiologist might or might not have access to the initial report, because the patient might forget to bring it or because it might not yet have been uploaded to the electronic medical record system. Therefore, it might not always be possible to compare interpretations. The second-opinion report is then incorporated into the permanent medical record for the patient at the receiving institution and is reviewed at the multidisciplinary meetings at which the management of cancer patients is discussed. It is probable that this procedure has also been followed at a number of institutions and cancer centers, because of the belief that radiologists who specialize in gynecologic oncology at tertiary care centers have incremental and essential expertise that can provide a more accurate diagnosis and better assessment of the extent of disease, both of which are crucial for planning the most appropriate treatment^(^^2^^)^. In addition, previous studies have demonstrated significant discrepancies between reports, which could significantly change the treatment given^(^^3^^)^.

In this study, we chose to focus on the evaluation of endometrial cancer, not only because it is the most prevalent gynecologic cancer in women but also because the majority of cases manifest at an early-stage, patients with endometrial cancer therefore requiring comprehensive preoperative staging. Endometrial cancer is illustrative because we can rely on histopathology to determine which of two interpretations is more accurate^(^^4^^)^.

The purpose of this study was to determine whether there are substantive discrepancies between initial and second-opinion interpretations, as well as to evaluate the added value of second interpretations provided by radiologists specializing in gynecologic oncology, concerning magnetic resonance imaging (MRI) scans obtained for endometrial cancer staging.

## MATERIALS AND METHODS

This was a retrospective, single-center study that was approved by the local institutional review board. We reviewed 232 MRI examinations performed and interpreted at outside radiology facilities for the staging of endometrial cancer, the provisional diagnosis having been based on patient symptoms or reports of previous ultrasound examinations. The MRI scans were submitted to a radiologist specializing in gynecologic oncology, for a second opinion, between January 1, 2013 and December 31, 2015. All outside reports were reviewed by one of the two radiologists specializing in gynecologic oncology at our facility (with 4 and 17 years of experience, respectively), both of whom consistently participated in weekly multidisciplinary meetings in their field. To ensure that access to the information was the same for the initial and second reports, we excluded cases in which additional imaging examinations were performed or additional tissue samples were collected.

**Figure 1:**
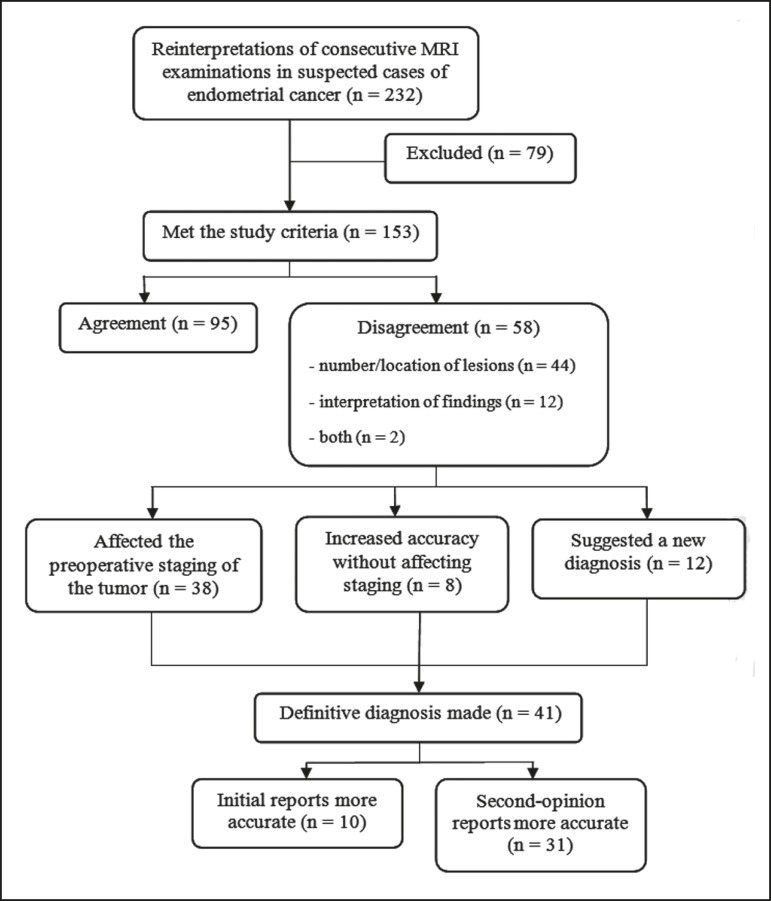
Flow chart of the study design.

We initiated the study after all of the reports and images from the referring institutions had been imported into our picture archiving and communication system. We excluded 52 examinations in which there was no outside report for comparison. An additional 27 examinations failed to meet the inclusion criteria. Therefore, the final sample comprised 153 examinations. A flow chart of the study design is shown in Figure 1.

### Agreement versus disagreement

The initial and second-opinion radiology reports were compared and categorized in terms of agreement or disagreement. Disagreement was defined as discordances regarding the final suggested diagnosis or discrepancies regarding clinically relevant imaging findings (minor aspects such as hepatic steatosis and renal cysts were not taken in consideration).

Disagreement was identified in 58 cases, all of which were carefully reviewed. The discrepancies were graded according to the number and location of lesions (e.g., invasion of the cervix, bladder, or rectum, or enlarged lymph nodes), as well as according to the interpretation of findings (e.g., the identification of the origin of the mass). If the initial report indicated endometrial cancer, we evaluated disagreements regarding the following: the depth of myometrial invasion; cervical stromal invasion; pelvic or para-aortic lymph node enlargement; local or regional spread (to the uterine serosa, parametrium, adnexa, vagina, bladder, or rectum); and distant metastases (intra-abdominal metastases or metastases to inguinal lymph nodes).

By consulting the medical records, particularly the decisions made in multidisciplinary meetings, in view of the International Federation of Gynecology and Obstetrics Staging System for Endometrial Cancer^(^^5^^)^, as outlined in Table 1, we determined whether the second-opinion interpretations affected the preoperative tumor staging, provided information that was more accurate without affecting the preoperative staging, or suggested a new diagnosis of cancer. Available histopathology reports provided the definitive diagnosis.

**Table 1:** Revised (2009) International Federation of Gynecology and Obstetrics Staging System for Endometrial Cancer.

Stage	Finding(s)
I*	Tumor confined to the corpus uteri
IA*	No invasion or invasion of less than half of the myometrium
IB*	Invasion of half or more than half of the myometrium
II*	Tumor invading the cervical stroma but not extending beyond the uterus^†^
III*	Local and/or regional spread of the tumor
IIIA*	Tumor invading the serosa of the corpus uteri and/or adnexa^‡^
IIIB*	Vaginal and/or parametrial involvement^‡^
IIIC*	Metastases to pelvic and/or para-aortic lymph nodes^‡^
IIIC1*	Positive pelvic lymph nodes
IIIC2*	Positive para-aortic lymph nodes with or without positive pelvic lymph nodes
IV*	Tumor invading the bladder and/or bowel mucosa, with or with-out distant metastases
IVA*	Tumor invading the bladder and/or bowel mucosa
IVB*	Distant metastases, including intra-abdominal metastases and/ or inguinal lymph nodes

* G1, G2, or G3.

† Endocervical glandular involvement only should now be considered indicative of stage I rather than stage II.

‡ Positive cytology should be reported separately and does not alter the stage.

The technical aspects of MRI were also taken into consideration. We evaluated image quality (artifacts, field-of-view, slice thickness, and magnet strength) as well as the pertinence of the sequences obtained, according to the guidelines established by the European Society of Urogenital Radiology for the use of MRI in the staging of endometrial cancer^(^^6^^)^.

### Statistical analysis

Confidence intervals (CIs) for all rates were calculated by Wilson score interval with continuity correction^(^^7^^)^. All statistical computations were performed by using the R programming language (version 3.1.2; R Foundation for Statistical Computing, Vienna, Austria) for Microsoft Windows^(^^8^^)^.

## RESULTS

**Figure 2:**
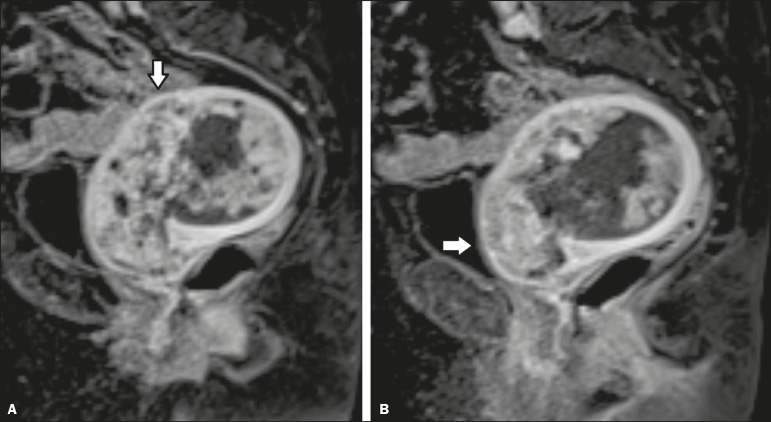
MRI scan from an outside facility showing the pelvis of a 63-year-old female with endometrial cancer. Gadolinium-enhanced fat-suppressed sagittal T1-weighted images show cancer invading more than half of the myometrium (**A**) and the cervical stroma (**B**). The initial report described an endometrial tumor invading less than half of the myometrium. Subsequent histopathology confirmed the findings of the second-opinion interpretation.

Among the 153 sets of MRI reports evaluated, congruence between the initial interpretation made at the referring institutions and the second-opinion interpretation made at the tertiary care center was seen in 95 (62.1%; 95% CI: 53.9-69.7), disagreement being seen in the remaining 58 (37.9%; 95% CI: 30.3-46.1). In the 58 cases of disagreement, the time between the initial report and the second-opinion report ranged from 1 to 15 weeks (median: 5 weeks). Among those 58 cases, discrepancies concerning the number and location of lesions alone were seen in 44 of those 58 cases (75.9%; 95% CI: 62.5-85.7), whereas discrepancies concerning the interpretation of findings alone were seen in 12 (20.7%; 95% CI: 11.6-33.7) and discrepancies concerning both aspects were seen in 2 (3.4%; 95% CI: 0.6-13.0). Among the 46 cases in which there was disagreement regarding the number and location of lesions, the discrepancies were related to the depth of myometrial invasion in 23 (50.0%), cervical stromal invasion in 10 (21.7%), pelvic or para-aortic lymph nodes in 6 (13.0%), local or regional spread, as depicted in Figure 2, in 18 (39.1%), and distant metastases in 5 (11.3%).

**Figure 3:**
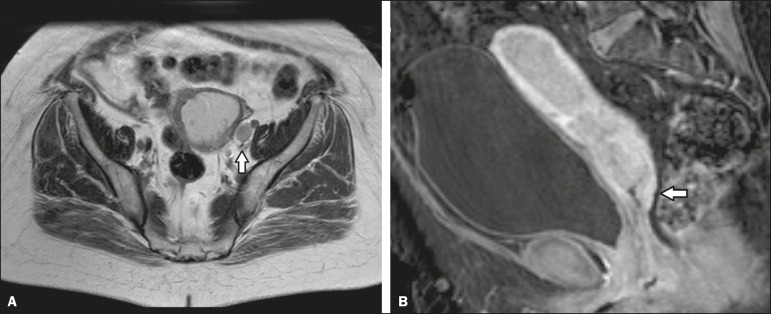
Axial T2-weighted image (**A**) and gadolinium-enhanced fat-suppressed sagittal T1-weighted image (**B**) from an outside facility. Neither the enlargement of the left external iliac lymph node nor the invasion of the upper posterior third of the vagina was reported in the initial interpretation of the MRI scans of this patient with endometrial cancer invading more than half of the myometrium and the cervical stroma. Although those findings led to preoperative upstaging, the decision made by the multidisciplinary board was that radiotherapy would have been the first approach to treatment in either case. Therefore, the second-opinion report did not affect the management in this particular case.

The second-opinion reports affected the preoperative tumor staging in 38 of the 153 cases (24.8%; 95% CI: 24.8-32.2), provided information that was more accurate without affecting the preoperative staging in 8 (5.2%; 95% CI: 5.2-9.9), and suggested a new cancer diagnosis in 12 (7.8%; 95% CI: 7.8-13.2). We believe that the second-opinion report had an impact on patient care in 37 cases (24.2%; 95% CI: 17.8-31.9), as illustrated by the case depicted in Figure 3.

During the study period, some of the patients died, others declined surgery, and others decided to undergo treatment at another facility. Therefore, the definitive diagnosis was made in only 41 of the 58 cases in which there was disagreement between the initial and second-opinion MRI reports. In 31 of those 41 cases, histopathological analyses demonstrated that the second-opinion interpretations were more accurate in predicting the final diagnosis (75.6%; 95% CI: 59.4-87.1), and in 10 cases the second-opinion interpretations were more accurate in predicting the initial diagnosis (24.4%; 95% CI: 12.9-40.6).

Moreover, discrepancies were more frequent in patients with advanced disease than in those in early-stage. In the 95 studies of the agreement group, 81 (85.3%) were early-stage and 14 (14.7%) were advanced disease, compared to the 58 cases of the disagreement group, where 37 (63.8%) were early-stage and 21 (36.2%) were advanced disease. The quality of the MRI scans obtained at the referring facilities was considered adequate in only 31 of the 58 cases in which there was disagreement (53.5%; 95% CI: 40.0-66.5). In addition, when determining the pertinence of the sequences obtained, we found that at least one key pulse sequence was absent in 35 of those cases (60.3%; 95% CI: 46.6-72.7) and that unnecessary sequences were obtained in 28 (48.3%; 95% CI: 35.1-61.6). The MRI sequences that were most often missing were multiphase contrast-enhanced pulse sequences, in 32 cases (55.2%; 95% CI: 41.6-68.0) and T2-weighted axial oblique (perpendicular) or coronal oblique (parallel) sequences of the uterine cavity in 23 (39.7%; 95% CI: 27.3-53.4).

## DISCUSSION

Although there have been a number of studies regarding second-opinion interpretations of imaging examinations^(^^1^^)^, few have addressed gynecologic oncology reports. In addition, we are unaware of any studies specifically focusing on the use of MRI in the evaluation of presumed endometrial carcinoma.

Endometrial cancer is the fourth most common malignancy in women^(^^9^^)^. Because knowledge of the extent of the tumor determines prognosis and appropriate treatment^(^^10^^)^, it is crucial that all radiologists reporting on MRI examinations of the female pelvis be familiar with the presentation of endometrial cancer and its routes of dissemination that allow accurate identification of key imaging findings, thereby providing the clinician with a useful tool to inform decisions regarding the most appropriate treatment.

Approximately 80% of all cases of endometrial cancer are diagnosed at stage I, probably due to the early development of signs and symptoms such as vaginal spotting or bleeding^(^^10,11^^)^. Five-year survival rates range from 96% for cases diagnosed at stage I to 25% for those diagnosed at stage IV^(^^4^^)^. The prognosis relies on numerous factors, such as the stage, the depth of myometrial invasion, cervical stromal invasion, lymphovascular invasion, lymph node invasion, and histologic grade^(^^12^^)^. Although MRI is not recommended as a screening procedure in the diagnosis of endometrial carcinoma, it has proven to be an important tool for the staging of known cancer, being particularly useful in the preoperative assessment because it allows pre-treatment risk stratification, thus allowing the surgical approach to be individualized and radical surgery to be avoided for patients who are at low risk^(^^12-14^^)^.

In our study, the interpretations of the referring radiologists showed substantial disagreement with those of the radiologists specializing in gynecologic oncology at the receiving facility in nearly one third of the cases. Discrepancies were more common among the patients with advanced-stage endometrial cancer than among those with an early stage of the disease, which is expected because of the increased complexity of interpreting the findings. However, given that diagnosis of the disease in its early stages is far more common^(^^10,11^^)^, it is not surprising that most of the patients in our sample had been diagnosed at stage I or II and that the most common discrepant finding was related to the depth of myometrial invasion.

In the present study, the final diagnosis was in agreement with the second-opinion interpretation in the majority of the cases in which a final diagnosis was made. In the majority of the cases in which the final diagnosis was in agreement with the initial interpretation, the discrepancy was due to preoperative upstaging of the cancer with regard to the depth of myometrial invasion or to cervical stromal invasion. Considering that the waiting time for elective surgery is up to 4 months and that the definitive diagnosis was determined mostly by histopathology reports of surgical specimens, we believe that, by the time of the surgery, some of these tumors, especially those of the higher-grade subtypes, might have grown and spread enough to be categorized as they were in the initial report.

Our results are in agreement with those of other studies in which investigators showed that the reinterpretation of imaging examinations by radiologists with a subspecialization in a relevant field can have a positive effect on the management of cancer patients. Gollub et al.^(^^15^^)^ analyzed 213 whole-body CT scans of patients with proven malignancy submitted for a second-opinion review at a tertiary care center, finding a considerable (37%) rate of disagreement in interpretation and an actual change in patient care in 3% of the cases. Loevner et al.^(^^1^^)^ reported a significant (41%) rate of major discrepancies between the initial and second-opinion interpretations, the latter leading to a change in management and prognosis in over 95% of the cases evaluated at a head and neck cancer center. In a retrospective study performed at a pediatric hospital, Eakins et al.^(^^16^^)^ found a 41.8% rate of disagreement between the initial and second-opinion interpretations. The authors found that, among the patients evaluated for a definitive diagnosis, the second-opinion interpretations were more accurate than were the initial interpretations in 90.2%. Zan et al.^(^^3^^)^ reported discrepancies between initial and second-opinion neuroradiology interpretations in 347 (7.7%) of the 4534 examinations evaluated, the second-opinion interpretation being found to be more accurate in 84% of the cases.

Focusing on the second-opinion interpretation of MRI scans by radiologists specializing in gynecologic oncology, Lakhman et al.^(^^2^^)^ reviewed 469 MRI examinations, reporting disagreement between the initial and second-opinion interpretations in 38.6%, the latter potentially affecting patient management in more than 20%. The authors also found that the second-opinion interpretations were more accurate than were the initial interpretations in 83% of the cases, with clinically relevant inconsistencies. In a nationwide audit in the United Kingdom, Duncan et al.^(^^9^^)^ evaluated the accuracy of MRI in the staging of endometrial cancer and suggested that the evaluation of a higher number of cases can improve the performance of MRI in determining myometrial invasion and, to a lesser extent, cervical stromal invasion.

Our study has several limitations. First, we evaluated only MRI reports in which a second-opinion interpretation was officially requested, which might not have been totally random. Second, the classification and subclassification of disagreements require subjective judgments and involve interobserver variability, which could account for some of the discrepancies observed. Finally, there is also inherent subjectivity in the determination of whether disagreement could lead to a change in management, particularly in the small number of cases in which there was little detailed knowledge of the patient and the treatment decision tree was poorly described.

In conclusion, our findings support the premise that the second-opinion interpretations of imaging examinations by radiologists subspecializing in a relevant field at a tertiary care center provide added value. Second-opinion consultations can improve the accuracy of the diagnosis, staging, and management of cancer, potentially playing an important role from a financial perspective, because they can allow unnecessary procedures and examinations to be avoided. Increased expertise and subspecialty training should be encouraged in order to improve the medical care provided to patients.
